# Dark side of the principles of non-discrimination and proportionality: the case of mandatory vaccination

**DOI:** 10.1136/jme-2023-108998

**Published:** 2023-08-16

**Authors:** Filip Horák, Jakub Dienstbier

**Affiliations:** 1 Constitutional Law, Charles University Faculty of Law, Praha, Czech Republic

**Keywords:** Human Rights, Public Policy, Ethics- Medical, Right to Health

## Abstract

Deciding the conflict between various rights and interests, especially in medical ethics where health and lives are in question, has significant challenges, and to obtain appropriate outcomes, it is necessary to properly apply the principles of non-discrimination and proportionality. Using the example of mandatory vaccination policies, we show that this task becomes even more difficult when these principles lead us to counterintuitive and paradoxical results. Although the general purpose of these principles is to ensure that decisions and policies seek the highest and broadest possible enjoyment of rights for all (ie, the least restrictive solution), they achieve the complete opposite when applied to mandatory vaccination policies. To highlight and explain these paradoxical results, we present a typology of fifteen hypothetical mandatory vaccination policies containing various degrees of restriction and apply well-established non-discrimination and proportionality tests from constitutional law to each. We argue that mandatory vaccination policies exhibit two characteristics, namely the non-linear relationship between their general purposes and specific goals and the involvement of life and health, suggesting that more restrictive policies should prevail even though less restrictive policies might fail these tests. Using clearly structured and rigorous methodology from constitutional law, the proposed approach delivers a fresh view on the core ethical principles of non-discrimination and proportionality and a potentially useful tool in helping resolve also other challenges encountered in medical ethics beyond mandatory vaccination policies.

## Introduction

 Medical decision-making, whether concerning individual patients or public health, faces inherent challenges in balancing competing ethical considerations. The rights to health and life must be harmonised with bodily integrity and personal autonomy. Achieving this delicate equilibrium necessitates the application of two fundamental ethical principles: non-discrimination and proportionality.^1^,^3^ Notably, these principles are not exclusive to medical ethics but are also vital components of constitutional law, frequently enshrined in national constitutions and routinely used by courts to guide decision-making. In the domains of both medical ethics and constitutional law, these principles guide us in formulating and adopting decisions and policies which are the least invasive to human rights and do not arbitrarily differentiate between individuals. In other words, the purpose of these principles is to ensure that every decision seeks the highest and broadest possible enjoyment of rights for all. This is often interpreted rather straightforwardly in that less restrictive policies should be always preferred^3^,^5^ because they do not interfere with individual rights as extensively as more restrictive policies.

In this study, we use the example of mandatory vaccination policies (MVPs) to show that this is not always true. MVPs are an ideal example since they differ from ordinary public policies in two important aspects.

First, the relationship between the general purpose of MVPs (ie, the protection of individuals and society by preventing major disease outbreaks) and their specific goal (ie, maximising the vaccination rate in the population) is non-linear because the general purpose cannot be effectively achieved until the vaccination rate reaches the required threshold, generally referred to as herd immunity. Consequently, MVPs below a certain level of restriction might not to be able to achieve their intended effects in a given population and therefore should be replaced with more restrictive alternatives. Second, because the purpose of an MVP is to protect the health and lives of individuals, it is desirable to approach them from the perspective of positive constitutionalism and thus shift the burden of justification so that less restrictive MVPs must provide justification that they do not disproportionately jeopardise the rights to health and life compared with their more restrictive alternatives.

Our argument asserts that due to these unique aspects, adherence to the non-discrimination and proportionality principles can lead to counterintuitive or paradoxical outcomes, steering public institutions toward adopting more restrictive MVPs rather than the expected less restrictive ones. To prove this point, we first discuss our preliminary considerations and offer a typology of hypothetical MVPs for adoption by health authorities (chapter 1) and then examine the individual MVP types using well-grounded and robust non-discrimination (chapter 2) and proportionality (chapter 3) tests from constitutional law. This method (thought experiment) allows us to (A) demonstrate and describe these paradoxical results, (B) identify and explain the factors which cause them, (C) argue that under certain circumstances less restrictive vaccination policies should be rejected as disproportionate or discriminatory whereas the more restrictive policies should prevail and (D) provide a novel and useful constitutional legal perspective for medical ethics and a tool for more rigorously analysing these two ethical principles and their application.

### Preliminary considerations and a typology of MVPs

To achieve the aims of the study, we limited the scope of examined vaccination policies. First, we only included vaccines for which the principles of non-discrimination and proportionality lead to the paradoxical results that we want to analyse. A common characteristic of these vaccines is that they represent the only effective and viable alternative in protecting individuals' lives and health while also enabling the normal (ie, no emergency quarantine, etc) functioning of the country and society.^6^ More precisely, to be included in our study a vaccine needs to protect against outbreaks of (A) highly contagious and (B) serious diseases which are (C) not easily curable or prevented by other means. The included vaccines also need to be (D) sufficiently effective in the sense that they are able to achieve herd immunity in the population and (E) save, that is, approved by national and international authorities as being de lege artis.

Second, since non-MVPs do not interfere with the rights of individuals, they can be neither discriminatory nor disproportionate. Therefore, if non-mandatory policies suffice in a given society to achieve the required vaccination rate, they may indeed represent ideal solutions. In our study, we exclusively examine the scenario where MVPs are required because a significant portion of the population refuses voluntary vaccination.^7^ As no vaccine provides 100% protection, failure to adopt a MVP in such a scenario would then put the lives and health of all individuals at risk. However, these individuals are endowed with fundamental rights to life and health, which are (like any other individual rights) reflected in the obligations of others. These obligations can manifest either directly, such as the obligation of other people not to gun-shoot someone or indirectly (ie, through the government and its policies). An example of an indirect effect might concern accidentally running someone over with a car. Obviously, people cannot be obliged not to have car accidents. However, the government bears a positive obligation, derived from the rights to life and health, to adopt a sufficiently effective policy preventing car accidents from happening too frequently. Such a policy may entail a range of more or less restrictive obligations, such as requiring valid driving licenses, prohibiting intoxication while driving, regular vehicle inspections, observing speed limits and more. Ultimately, it does not matter whether obligations derived from the rights to life and health are direct or indirect. The core issue discussed in this article pertains to the restrictiveness of such obligations (ie, the extent of interference with individual rights) and whether these obligations can be justified (ie, whether they are in accordance with the principles of non-discrimination and proportionality).

**Figure 1:**
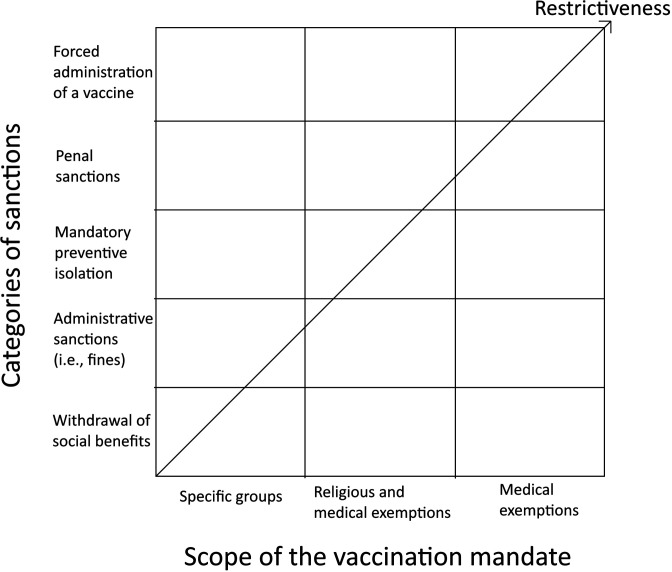
Typology of mandatory vaccination policies.

Hence, returning to highly contagious, serious and not easily curable diseases, we argue that if non-MVP does not suffice in a given society to achieve the required vaccination rate, the government has a positive obligation derived from the rights to life and health of its citizens to adopt one of the possible MVPs provided that it is non-discriminatory and proportionate. To systematically analyse these hypothetical MVPs from the perspective of non-discrimination and proportionality, we created a typology based on two criteria: the scope of the vaccination mandate (three included options) and the stringency of the sanction for choosing not to be vaccinated (five included options).^8^ The resulting typology contains 15 hypothetical MVP types (figure 1).

The narrowest (and hence also least restrictive) scope that we included is the vaccination mandate for specific citizen groups (eg, children, healthcare workers, elderly). The other two are broader because they are based on a general vaccination mandate with some exemptions (ie, medical and religious or philosophical exemptions in the second case and solely medical exemptions in the third and broadest case). We excluded the general vaccination mandate without any exemptions since it would in fact force medical personnel to administer a vaccine, even if it is known that it is highly likely to cause severe harm or even death to an individual with contraindications. This situation would not only be absurd, but also contrary to the very essence of medical ethics, because there is a significant difference between accepting a certain (limited) risk of vaccines’ side effects on individuals without previous episodes that suggest contraindications (which can then be weighed against the risks of not achieving a sufficient vaccination rate in the population; see chapters 2 and 3), and knowingly (intentionally) harming an individual with already identified contraindications.

In addition to the three different scopes of vaccination mandate, we included five sanction categories for choosing not to be vaccinated. We assume that each category of sanctions is more stringent (and thus more restrictive) than the previous. The first category (ie, withdrawal of social benefits) contains sanctions which interfere with social (third generation) rights (eg, the right to various allowances or the right to free education in public schools or kindergartens). These rights, for their mainly political nature and dependency on the financial and budgetary situation of the government (and society), are often afforded a lesser degree of judicial protection than first and second generation rights.^9^ Hence, we argue that interference with these rights should also be considered principally less restrictive.

The general instrument of the second category of sanctions (ie, administrative sanctions) is a fine. These sanctions thus interfere solely with property rights. Compared with the previous category, these sanctions are more restrictive since they are based on taking already earned material goods away rather than refusing to give them. Nevertheless, they still target material goods, and unless they interfere extensively with basic existential needs^10^ should be considered less restrictive than a sanction interfering with other rights.

The third category of sanctions (ie, mandatory preventive isolation) includes, for example, prohibition of using public transport, entering the workplace or eating in restaurants. These sanctions interfere with rights and freedoms (eg, freedom of movement, the right to work or right to privacy) of great importance in a person’s life and development and are thus undoubtedly more restrictive than the previous sanctions.

The fourth category (ie, penal sanctions) is most often represented by the sanction of imprisonment, which is even more restrictive than the previous category since it interferes with the very core of an individual’s personal liberty.

The final and most restrictive category of sanctions (ie, forced administration of a vaccine) interferes with fundamental personal autonomy, bodily integrity and the rights to health and life. Individuals are deprived of the free choice whether to be vaccinated (ie, a free decision concerning one’s own body); they are forced to undergo the administration of a vaccine and expose themselves to its possible side effects.

### The principle of non-discrimination

To correctly apply the principle of non-discrimination^11^ and examine whether a given MVP is discriminatory, we apply the antidiscrimination test, which generally consists of four steps.^11 12^

First, we consider whether people subject to an MVP and who are otherwise in a roughly equal situation or position (ie, comparable individuals) are treated or impacted differently (ie, a difference in treatment).

Second, we investigate whether the difference in treatment is based on criteria which divide the population into at least two groups and whether such criteria directly target personal characteristics (eg, physical or mental condition, nationality, age, gender, sexual orientation) or actions or the results of previous actions such as criminal record, university diploma, mountain climbing, eating fast food frequently, smoking, etc (ie, differentiating criteria). Some of the criteria directly concerning individuals (especially those which may interfere with human dignity, such as nationality, ethnicity, race) are also considered ‘suspicious’ by some courts,^13 14^ and if applied, need very compelling reasons to be justified (see fourth step).

Third, we examine whether the difference in treatment disadvantages members of one group over members of other groups defined according to the same criteria (ie, relative disadvantage), whether these people are disadvantaged because they are members of that specific group (ie, causality), and whether the disadvantage is not merely an inconvenience (ie, severity of interference).

Fourth, we consider whether the relative disadvantage can be proportionately justified with respect to the legitimate purpose of the examined MVP (ie, proportionate justification). If any MVP shows a potentially discriminatory character in the first three steps of the test and cannot be proportionately justified in the fourth, it should be rejected since it violates the principle of non-discrimination.^15^

### Comparable individuals and differences in treatment

Several MVPs treat or affect comparable individuals differently. In fact, looking at the scope of vaccination mandates, no MVP applies to all people without exception. Hence, some individuals are always treated differently because they are required to be vaccinated whereas others are not. However, each unvaccinated individual who has the potential to be infected with a disease and further spread it poses a risk to public health and the rights to life and health of other people.^16^ From this perspective, all individuals (except perhaps those who are 100% immune to the disease) are roughly in an equal position. Consequently, all the aforementioned policies are potentially discriminatory and should be further investigated in the following steps.

Besides the scope of the vaccination mandate, the category of sanctions prescribing mandatory preventive isolation is also problematic. These sanctions are unique for two reasons. First, they are not one-time sanctions punishing the refusal to be vaccinated. Instead, they are potentially infinite since they apply to a person as long as she/he is not vaccinated. Second, those who execute these sanctions are primarily private persons rather than public officials or institutions (eg, public transport operators who do not permit unvaccinated passengers to board their vehicles, employers who prevent unvaccinated employees from entering the workplace, or restaurateurs who refuse to serve unvaccinated customers). Hence, this category of sanctions in fact distinguishes a group of citizens who, although still citizens and thus in an equal position as others, are prevented from fully enjoying certain fundamental rights. It is worth noting that the difference in treatment here lies in the sanction for not being vaccinated rather than the vaccination mandate itself. Hence, equal position relates to citizenship and the enjoyment of rights rather than the risks an unvaccinated person poses to public health and the rights of others. The latter can only serve as a proportionate justification in the final step of the test. Therefore, we argue that MVPs prescribing mandatory preventive isolation are also potentially discriminatory and should be further investigated in the following steps.

### Differentiating criteria

Several differentiating criteria can be identified in further analysis of MVP types which treat comparable individuals differently. Regarding the scope of the vaccination mandate, the narrowest MVPs applicable to specific groups of citizens generally differentiate age (eg, children), health condition (eg, people vulnerable to the disease) or profession (eg, emergency personnel). The broader MVPs which exempt some citizens from an otherwise general vaccination mandate differentiate health conditions (ie, contraindication)^17^ and in the case of the narrower of the two, also religious faith (or philosophical and ethical beliefs).^18^ Finally, vaccination status itself becomes a differentiating criterion under the sanction of mandatory preventive isolation. We should note that MVPs in liberal democracies rarely use the dignity-interfering suspicious criteria, although one exception might be religious faith.

### Relative disadvantage, causality and severity of interference

To be regarded as discriminatory, a differentiating treatment must result in a person’s disadvantage of a certain degree (ie, it must cause negative interference with a person’s rights or interests which are not a mere inconvenience).^11^ Unfortunately, the intensity of the interference is sometimes very difficult to gauge. Four groups of disadvantages can be distinguished from the analysed MVP types.

The first three groups, namely (A) interference with the rights to health and life due to the vaccine (ie, possible side effects), (B) interference with the rights to personal autonomy and bodily integrity (ie, a person may not freely decide whether to be administered a foreign substance into his or her body) and (C) interference with freedom of religion or consciousness by not permitting religious exemption (ie, people are forced to undergo something they may consider unethical or religiously problematic), are linked to the vaccination mandate and its scope.^19^ In other words, the people who are obliged to be vaccinated may suffer these interferences whereas others do not. The final group of disadvantages stems from the specifics of mandatory preventive isolation for unvaccinated citizens and potentially contains (D) interference with a range of rights linked to an individual’s autonomy, such as freedom of movement, the right to work and the right to privacy in a broader sense.^20^

Regarding the causality between differential treatment and interference, it is evident that all the examined MVP types interfere with an individual’s rights because all individuals belong to groups distinguished according to the criteria described above. It is so because the policies explicitly apply these criteria to determine who should be vaccinated or sanctioned and who should not. Furthermore, since each of the above-mentioned disadvantages interferes with fundamental rights, they cannot be understood as mere inconveniences.

### Proportionate Justification

Since all the mentioned MVP types treat comparable individuals differently according to personal (in one case also suspicious) criteria and may significantly interfere with the rights of the disadvantaged group, the final decision on whether these policies are discriminatory is determined from proportionate justification, the final and critical step of the non-discrimination test.

This step links the principle of non-discrimination to the proportionality test,^15^ where we ask whether reasons that proportionately justify interference with the rights of the disadvantaged group exist. In other words, we weigh the probability and severity of the interference with the benefits (with respect to the legitimate purpose of the given policy) of treating comparable individuals differently.^15^ It follows that if the differentiating treatment has no benefits compared with a non-differentiating treatment, it should automatically fail and be rejected as discriminatory. Conversely, if discriminatory treatment has any benefits, then we need to perform a proportionality test to get the final results. The specifics of proportionate justification for all the studied situations of differentiating treatment are elaborated in the next chapter.

### The principle of proportionality

The second important principle we examine and apply to outlined MVP types is proportionality which serves as a tool whereby conflicts between (A) various individual rights or (B) individual rights and governmental objectives are resolved in a way which minimises the interferences with all the conflicting entities.^21^ The application of this principle in individual cases involves three steps, preceded by identifying the interference with individual rights (‘step 0’). In the case of MVPs, interference stems from either differentiating treatment (chapter 2) or the sanctions for choosing not to be vaccinated, which we discuss below.

This chapter is structured according to the three steps of the proportionality test applied in constitutional law. If an MVP fails to pass any of the three steps (regardless of whether it is too restrictive or, on the contrary, too benevolent), we refer to it as disproportionate and argue that it should be rejected.

The first step investigates whether the examined MVPs are able to achieve (ie, suitability)^21^ objectives which can be considered legitimate (ie, legitimate purpose).^21^ For any governmental objective to be legitimate, it must pursue purposes which agree with the key principles and values that create the axiological core of a given society. From the legal perspective, these principles and values are explicitly enshrined in or can at least be implicitly derived from the constitution of the respective nation (eg, the protection of individual rights or crucial public interests).

In the second step, we examine whether a given MVP achieves legitimate purposes in the least restrictive manner. More specifically, we look for alternative policies which, in a given society, would achieve the same legitimate purposes with at least equal efficiency while also causing less interference with individual rights (ie, necessity).

Finally, in the third step, we directly weigh the benefits of a given suitable and necessary MVP with its costs in the form of interference with individual rights (ie, proportionality in a narrower sense). In this balancing exercise, the social significance of the rights and interests concerned, the intensity of interference with those rights, and the probability of such interference must be taken into account.^21^ An MVP may be considered proportionate in a narrower sense only when its benefits in a given society outweigh its costs. However, it is often impossible to establish beyond any reasonable doubt that the fundamental rights on one side of the balancing exercise outweigh those on the other. This raises the question of whose rights should prevail in cases of uncertainty (ie, who bears the burden of justification). Since we have adopted the perspective of so-called positive constitutionalism, we explain in the following sections why, in the case of MVPs, the right to health and life (ie, the right of every individual not to be exposed to the risk of infection from unvaccinated citizens) should be preferred.

It should be noted that it is sometimes argued that fundamental rights are incommensurable and that their weighting is therefore undesirable because it deprives them of a special status^22^ and reduces them to mere public interests.^23^ We disagree with this criticism, however, because rejecting the balancing exercise would effectively result in an inability to resolve any dispute between two or more conflicting fundamental rights (ie, denegatio iustitiae). Moreover, it is the proportionality test that allows us to understand fundamental rights as principles and to ensure that they are protected to the fullest extent possible in each individual case.^21 24 25^

### Legitimate purpose and suitability

The proportionality test begins with assessing the purpose of the studied MVP types. First, we excluded the protection of individuals against their own wishes, as several influential ethical theories contest the legitimacy of this purpose for its excessively paternalistic nature.^26 27^ The remaining legitimate purposes of MVPs can be divided into two groups: individual and collective. The individual purpose is to protect the individual rights of others to life and health.^19 28^ This is especially true for individuals who are not able to make informed decisions (children) or cannot be vaccinated because of an existing medical condition (contraindication).^16^ Moreover, almost none of the vaccines are 100% effective.^29^ Therefore, the lives and health of people who have already been vaccinated is an important factor, too. Collective purposes include the protection of public health, maintaining the stability of the healthcare system and the normal functioning of the economy, state and society.^30 31^ Individual and collective general purposes are intertwined. For example, a large-scale epidemic might overload healthcare facilities and thus significantly limit the availability of healthcare, even for patients with concerns unrelated to the epidemic.

Within these general, long-term strategic purposes, we can identify more specific goals. Leaving aside the ideal notion of complete eradication of the disease, which has been possible with one disease only (ie, smallpox), we are left with the goal of vaccinating 100% of the population. Even this goal is idealistic: the composition of population continuously changes, and it is not possible to gather all the relevant data which reflects the whole of reality.

**Figure 2:**
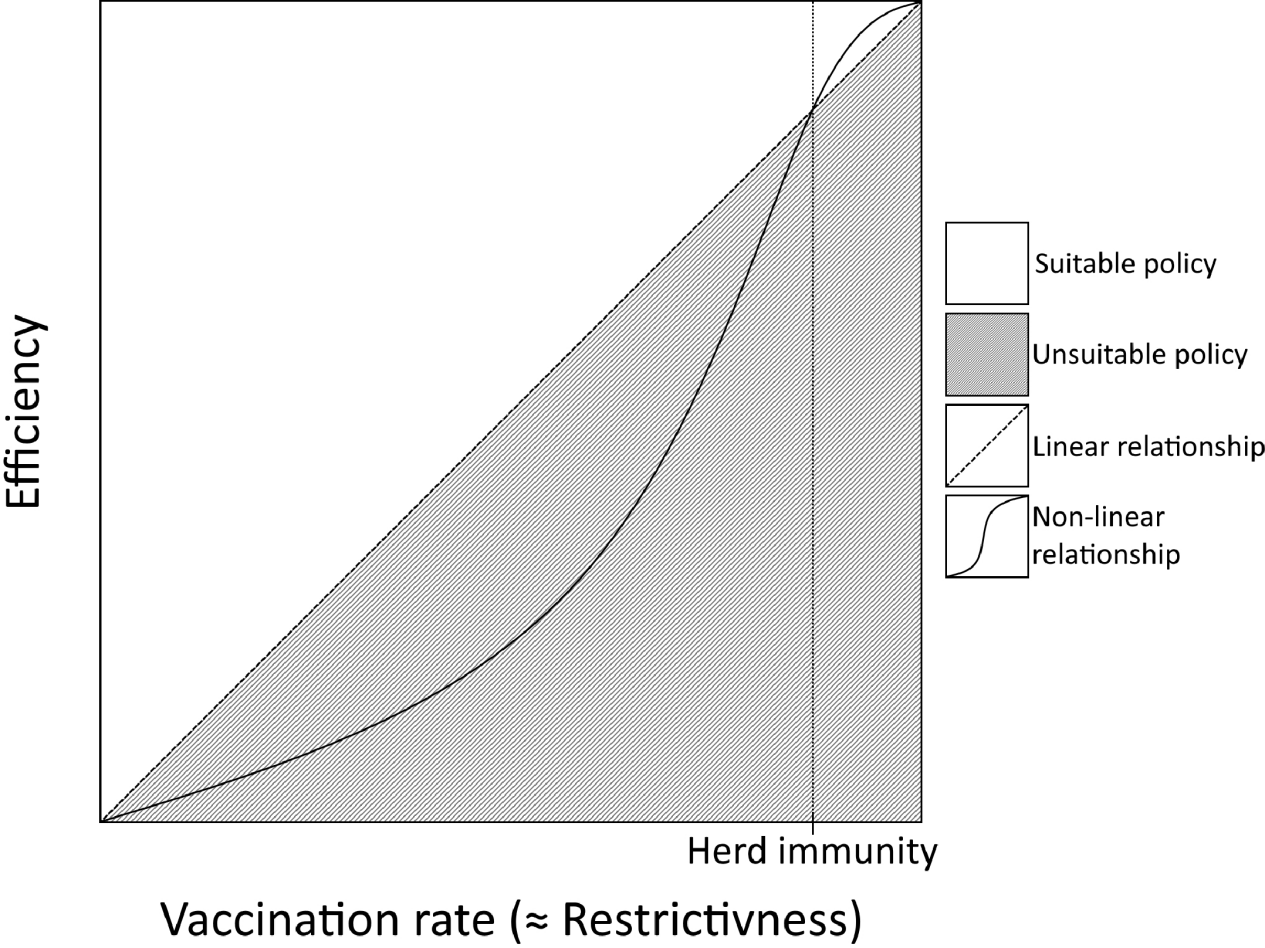
Non-linear relationship between the efficiency of protecting the general purposes of mandatory vaccination policies and the vaccination rate.

The only realistic goal is to vaccinate as many people in the population as possible. However, if we define a goal that may be achieved to various degree (and the greater degree we achieve the better), we assume that a linear relationship exists between this goal and the general purposes which justify it. This would mean that the efficiency of protecting the aforementioned general purposes increases in direct proportion to the vaccination rate in the population; however, this is sadly not true. If we truly want to protect the stability of health, economic, social and other systems and the rights of individuals to health and life, we must achieve a vaccination rate that prevents a large-scale epidemic of the disease. A vaccination rate such as this, generally referred to as herd immunity, is estimated at 70%–95% (depending on the disease).^32 33^ Hence, we argue that the relationship between the vaccination rate and the degree to which the general legitimate purposes are achieved is non-linear.^18^ Illustrated in figure 2, the efficiency of achieving legitimate purposes rises relatively slowly at first and increases more steeply as the vaccination rate matches herd immunity. After achieving this threshold, it rises again relatively slowly.

The non-linear relationship between the efficiency of protecting general purposes and the vaccination rate then requires redefining the specific legitimate goal of the MVP as maximisation of the vaccination rate in the population to achieve at least the level required for herd immunity. Considering this non-linear relationship, we can define the suitability of the examined MVP as the ability to achieve at least the vaccination rate required for herd immunity in the population.

Looking again at the MVP types identified as potentially discriminatory, we should reject vaccination mandates restricted to specific groups as unsuitable since these groups (even if 100% vaccinated) generally do not represent a sufficient portion of the population for herd immunity to be achieved. Differentiating treatment of this kind cannot be proportionately justified and should be rejected as discriminatory.

By contrast, the other two possibilities for determining the vaccination mandate’s scope cannot be a priori rejected as unsuitable since the portion of the population which cannot claim religious (or philosophical) or medical exception may still be sufficient for herd immunity. This is even truer if an MVP allows medical exemptions only. However, demographic characteristics in some societies might show otherwise.^34^ The suitability of these exemptions should, therefore, be assessed separately in each case.

The suitability of the remaining potentially discriminatory MVP types (ie, those applying the sanction of mandatory preventive isolation to non-vaccinated individuals) can be examined together with other sanction categories, because the specifics of this particular sanction given by its potentially discriminatory character are relevant only in the third step of the proportionality test.

We have already explained why each of the five sanction categories in the typology is more stringent (and restrictive) than the previous. Let us now assume that the efficiency of any MVP (ie, the percentage of the population compliant with the legal obligation to be vaccinated) increases with the restrictiveness of the sanctions because refusing vaccination becomes increasingly disadvantageous. This means that more restrictive sanction categories generally have a higher chance of achieving herd immunity than less restrictive ones. However, the reaction to greater restrictiveness in sanctions may vary significantly between societies, a topic which could be interesting for empirical research. In some societies, most individuals may already be persuaded by the least restrictive sanctions whereas in others, even penal sanctions may not be sufficient. Therefore, in terms of the suitability of the examined MVPs, no universally applicable conclusions can be drawn. We can, however, derive the following general rule: in each society, all sanction categories which are not able to persuade enough individuals to achieve herd immunity must be rejected as unsuitable and consequently disproportionate.

We can already observe that the non-linear relationship between the general purposes and specific goals of MVPs has completely shifted the outcomes of the suitability check. The narrower vaccination mandates and less stringent sanction categories are rejected as discriminatory or disproportionate whereas the broader mandates and more stringent categories (ie, the more restrictive MVP types) are suitable.

### Necessity

In the second step, we assess the necessity of the suitable MVP types (in a given society). Since the suitability check has already resolved the issue of the non-linear relationship between general purposes and specific goals and excluded the MVPs unable to achieve herd immunity in the population, we are now left with the original goal of vaccinating as many people in the population as possible. Hence, necessity can be defined as the absence of less restrictive but at least equally efficient alternative methods of maximising the vaccination rate.

In other words, we should reject any suitable MVP type as unnecessary only when its greater restrictiveness does not lead to any increase in the vaccination rate. This returns us to the assumption that more stringent sanctions generally lead to greater compliance and thus a higher vaccination rate. This is even truer for the scope of the vaccination mandate: the broader the scope (ie, the more people who are legally obliged to be vaccinated) the higher the vaccination rate we can assume in the population.

It follows that none of the suitable scopes of vaccination mandate (ie, general vaccination mandate with medical and religious exemptions or exclusively medical exemptions) and none of the suitable sanction categories (potentially all) can be a priori deemed unnecessary, because although each scope and each sanction is arguably more restrictive than the previous, it is also presumably more efficient in maximising the vaccination rate. Hence, both scopes and all sanction categories must be further investigated in the following step.

The results of the necessity check underline the observation that narrower scopes and less stringent sanctions have a higher probability of being rejected as unsuitable and thus also discriminatory or disproportionate whereas broader scopes and more stringent sanctions have a good chance of prevailing as suitable and necessary in a given society.

### Proportionality in a narrower sense

Moving to the final step of the proportionality test, a preliminary thought should be considered: although a weighting procedure appears to mathematically balance (marginal) benefits or other quantifiable elements, the opposite is true.^35^ The reasoning behind this step is strongly linked to the moral and political background of the individual arguments and the context of the given society.^21 35^

Any assessment of the weight of individual arguments is also strongly affected by the prior understanding of those performing the balancing exercise.^21^ In the case of MVPs, a potentially significant difference exists between the perspectives of medical professionals and those of (constitutional) lawyers. From a medical point of view, the protection of health and life is the default value, and hence, a medical professional may formulate the balancing question as follows: *Do the individual rights interfered with by the MVP outweigh the right to health and life protected by this policy?*.^36 37^ By contrast, constitutional law is traditionally based on the primacy of the free (or autonomous) individual before the government, whose regulatory power is controlled and limited.^38^ A constitutional lawyer may formulate the balancing question in quite the opposite way: *Does the protection of the right to health and life by the MVP outweigh the individual rights interfered with by this policy?* This difference is crucial since it determines who bears the burden of justification and whose rights win in the case of uncertainty.

However, the current trend in constitutional law is to strengthen argument based on the common good and positive obligations of the state (so-called positive or common good constitutionalism).^38 39^ This view does not understand the protection of individual rights only as a set of restrictions on public power; on the contrary, it expects government to perform active steps towards real implementation and the enjoyment of individual rights.^38 39^ This is especially desirable in medical domain where the health and lives of individuals are concerned.^40^ Even libertarians, who in the context of MVPs invoked the so-called ‘clean hands principle’, accepted this perspective.^27^ In our case, it is therefore legitimate to perform the balancing exercise based on the perspective of medical professionals, which means that in the case of uncertainty, the right to health and life wins (ie, the right of every individual not to be exposed to the risk of infection from unvaccinated citizens).

The balancing exercise for MVPs can be divided into three segments. First, we assess the proportionality (in a narrower sense) of the vaccination mandate itself. Second, we address the proportionality of the sanctions for not choosing not to be vaccinated. Finally, we examine the proportionality of potentially discriminatory MVP types.

Concerning the vaccination mandate itself, we weigh the protection of the health and lives of individuals and the protection of public health from a disease against the protection of health and life from the side effects of vaccination and the protection of personal autonomy and bodily integrity from vaccine administration.

The protection of health and life from dangerous, potentially lethal and highly contagious diseases easily outweighs the risks caused by the side effects of vaccines (if approved by competent authorities).^4 19^ These risks are generally minimised to negligible significance through a demanding vaccine testing process.^29^ Besides that, facilities administering vaccines should make honest efforts to reveal as many cases of contraindications as possible while considering the overall health status of vaccinated persons. Finally, a compensation system could be introduced to cover any permanent adverse consequences of vaccination.^30^ The burden that people under a vaccination mandate have to bear would, therefore, not be unreasonable or onerous.^41^

By contrast, the rejection of general immunisation of the population with the knowledge of its scientifically proven effectivity^42^ would pose a risk of significantly greater harm to both the health and lives of all individuals (especially vulnerable groups such as children, elderly, chronically ill or persons who cannot be vaccinated for health reasons)^41^ and the stability and normal functioning of society. Especially in recent decades, even if we leave COVID-19 pandemic aside, we have witnessed an increasing number of diseases that until recently were considered suppressed.^16^ Vaccination is a relatively inexpensive method of ensuring public health whereas other alternatives, such as super-robust hospital systems or isolation of unvaccinated people to deserted areas, are either unbearably expensive or simply absurd.

Finally, even though personal autonomy and bodily integrity are very important individual rights in contemporary liberal-democratic societies, being narrowly linked to the core ethical principles of human self-realisation, freedom and dignity,^43^ they still cannot outweigh the rights to health and life, which are also linked to human dignity since they are indispensable to living in decent conditions, free of pain and dehumanisation. A vaccination mandate interferes with personal autonomy and bodily integrity only briefly (during administration of the vaccine) and with negligible intensity (slight discomfort)^4^ and generally no relevant consequences in the ability to control one’s body and autonomously determine one’s own actions. In summary, we consider the vaccination mandate itself proportionate.

To continue our balancing exercise, we explained above that each sanctions category for choosing not to be vaccinated interferes with several individual rights which must always be added as arguments (weight) against the MVP types which apply that particular sanctions category. Nevertheless, we may encounter significant variation in each category. Administrative sanctions, for example, may impose a fine of €5 or €5000. Similarly, penal sanctions might range from a few months of house arrest to several years of imprisonment. It is therefore truly unfeasible to capture all the possible combinations of the balancing exercise in this article.

However, at a more abstract level, we could argue that interference caused by any of the sanction categories may not a priori outweigh the protection of health and life and the normal functioning of society as a whole. From a medical perspective, we might consider a less stringent sanctions category to be proportionate (in a given society) only if the benefits to the personal autonomies, bodily integrities and other rights of individuals who refuse vaccination clearly outweigh the interference with the rights to the health and lives of every other individual caused by the decrease in efficiency of the MVP. In other words, if applying a less stringent MVP means a significant decrease in vaccination rate in a given population, it should be rejected as disproportionate (in a narrower sense), provided that the more stringent MVP satisfies several safety, availability and procedural requirements. First, the sanctions are accompanied by sufficient information and educational campaigns explaining and justifying vaccination.^44^ Second, the vaccines are easily obtainable, information about the mandate and sanctions is generally known, and individuals are provided with sufficient time to comply with the mandate (ie, the possibility to obey).^45^ Third, individuals are granted procedural guarantees, including help from legal professionals, and judicial reviews of their cases^30^ Fourth, the parameters of the sanctions may not (under the standards of a given society) be absurd or draconian.^46^ Finally, the mandate or the sanctions must not be discriminatory.^37^

The final segment of the balancing exercise then examines the proportionality of the potentially discriminatory MVP types. First, we reiterate that any exemption from a vaccination mandate increases the risk of possible interference with the rights to the health and lives of other individuals. The right to equal treatment (ie, the right not to be discriminated against) for every other individual must also be added to the balancing exercise since exempt individuals may represent so-called ‘free riders’, whose benefit over others under the vaccination mandate is not having to bear any costs in receiving the vaccine or exposing themselves to possible side effects.^2 31^

However, exempt individuals also have specific rights which would suffer interference if exemption did not exist. For example, by administering the vaccine to people with contraindications, we might knowingly be causing a serious deterioration in their health or even endangering their lives. Essentially, we would be forcing the persons concerned to sacrifice themselves for the benefit of others. We can also expect that these people do not represent a significant portion of the population, therefore, the benefit from their contribution to achieving herd immunity would be rather small. It follows that exemption from a vaccination mandate due to contraindication is proportionate (and thus not discriminatory), provided that the risks the vaccine poses to the health and lives of these individuals are not significantly lower than the risks to the health and lives of others as a consequence of exempting these individuals.

A more complex problem is the question of exemption due to religious, ethical or philosophical beliefs. Individuals who insist that the vaccine itself, its ingredients or the processes of its manufacturing or testing are contrary to their religious, ethical or philosophical beliefs, might claim interference with their freedom of religion and consciousness.^47^ These types of exemption may, however, be considered proportionate (and not discriminatory) only when suitable (ie, when the existence of the exemption does not jeopardise achieving and maintaining herd immunity in a given society). Even in societies where the requirement of suitability is met, several arguments against this exemption still exist. First, interference with the freedom of religion and consciousness is only secondary since the essence of those rights (ie, to believe freely in what one wishes and to express one’s beliefs both privately and publicly) is not impacted.^19^ Second, it is morally and legally highly problematic to prefer any beliefs at the expense of the lives and health of other persons who do not share them.^48 49^ Third, the most important authorities of the major religions do not rule out a range of vaccines, including those that were developed or produced in a religiously questionable manner.^18^ Fourth, the fact that vaccination is the result of a general legal obligation on individuals rather than their active and wilful behaviour can potentially ease religiously or ethically driven remorse. All of these arguments are even strengthened in the case of an imminent or ongoing epidemic,^50 51^ where such exemptions could significantly contribute to the clustering of unvaccinated people.^52^ Hence, we argue that religious, ethical and philosophical exemptions must be (at least but arguably not only in secular societies) rejected as disproportionate in a narrower sense and thus discriminatory.^53^

Finally, in the previous chapter, we mentioned one more possibly discriminatory MVP type: those which apply sanctions of mandatory preventive isolation on the unvaccinated. Although at first glance it might appear to be concessional, protecting both the personal autonomy of those who do not want to be vaccinated and the lives and health of others, the opposite is true. By preventively isolating the unvaccinated, we create a special group of second-class citizens, denying them the enjoyment of benefits afforded to other members of society. Moreover, unless such persons are isolated absolutely (ie, gathered and detained in a deserted area), which is absurd, they would still pose a significant risk to the health and lives of others. In other words, the interferences with their rights caused by mandatory preventive isolation significantly outweigh the benefits to the rights to the health and life of others. Therefore, the sanction of mandatory preventive isolation should be also rejected as disproportionate and thus discriminatory.

In conclusion, the adopted perspective (preunderstanding) of medical professionals is another important factor which leads to the rejection (as disproportionate or discriminatory) of relatively less stringent sanctions and narrower vaccination mandates in favour of more stringent and broader mandates, provided that the MVP and vaccine satisfy aforementioned safety, availability and procedural requirements.

## Conclusions

**Figure 3:**
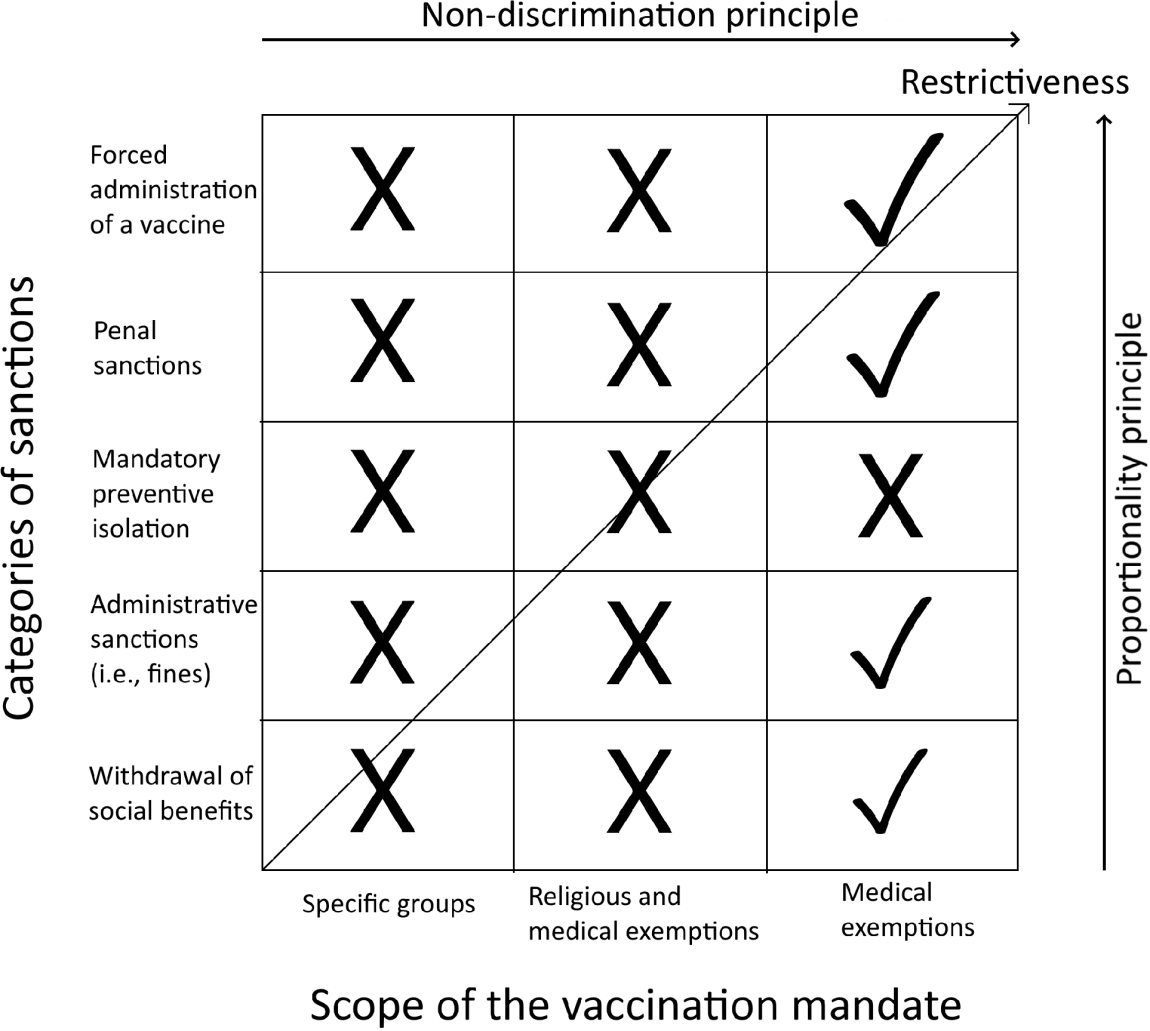
Application of the principles of non-discrimination and proportionality to the typology of mandatory vaccination policies.

After applying the traditional non-discrimination and proportionality tests from constitutional law to various hypothetical MVP types, we can verify the existence of paradoxical effects. Both analysed principles propel us away from the narrower scopes of vaccination mandates and less stringent sanctions for choosing not to be vaccinated towards broader scopes and more stringent sanctions (figure 3). The only scope we consider non-discriminatory is the broadest scope (ie, a general vaccination mandate with medical exemptions). Regarding the sanctions, each is potentially proportionate. The final result, however, depends on the demographic and cultural parameters of the given society. Considering these parameters, less stringent policies are generally more difficult to proportionately justify than more stringent policies. A specific case is the sanction of mandatory preventive isolation, which should be always rejected. The reason, however, is its discriminatory character rather than its general disproportionality.

We identified two key factors which create these counterintuitive (or paradoxical) results. The first factor is the non-linear relationship between the efficiency of the protection of the individual rights to health and life plus the stability and normal functioning of social systems (ie, the general legitimate purposes of the MVP) and the increase in vaccination rate in the population (ie, the more specific instrumental goal of the MVP). If the stringency of the sanctions is increased or the scope of the vaccination mandate is widened and consequently more individuals are persuaded to vaccinate, the efficiency of the MVP will remain relatively low until the vaccination rate required for herd immunity is reached. It follows that any less restrictive MVP which is not able to achieve herd immunity in a given society should be rejected as unsuitable whereas any more restrictive MVP which achieves the required vaccination rate should prevail.

The second factor is the choice between the two competing perspectives (preunderstandings). Unlike the traditional perspective of negative constitutionalism, positive constitutionalism, which better corresponds to the medical perspective, gives rise to positive obligations to not only respect but also actively protect, provide and fulfil individual rights as well as the common good. Such perspective also changes the narrative and results for proportionality (in a narrower sense). If this perspective is accepted, the state is expected to actively protect the rights to health and life of every (other) individual in cases of medically necessary vaccines by adopting an MVP type which is sufficiently efficient (ie, restrictive) in the given society. A less restrictive MVP is then justified only if the benefits to the rights of individuals refusing voluntary vaccination clearly and evidently outweigh the decrease of efficiency of the MVP (ie, the level of protection of the health and life of every other individual). Put differently, because of this factor, more stringent and broader MVPs can be justified more easily than less stringent, narrower MVPs.

In summary, the thought experiment presented in this article, based on the clearly structured and rigorous tests of constitutional law, casts a new light on the core ethical principles of non-discrimination and proportionality. However, we are aware that our results might appear relatively provocative and counterintuitive. We, therefore, highlight at least one important limitation in our conclusions concerning the sanction of forced vaccine administration. Even though such a sanction might be considered proportionate,^46^ in societies highly sensitive to violence, forced administration might be rejected as absolutely unacceptable and contrary to inviolable human dignity.^19 54^ In such societies, the principle of proportionality is a priori not applicable to this category of sanctions; discussion of this observation is beyond of the scope of this article, however.

## Data Availability

Data sharing not applicable as no datasets generated and/or analysed for this study.
